# Functional differences between microglia and monocytes after ischemic stroke

**DOI:** 10.1186/s12974-015-0329-1

**Published:** 2015-05-29

**Authors:** Rodney M. Ritzel, Anita R. Patel, Jeremy M. Grenier, Joshua Crapser, Rajkumar Verma, Evan R. Jellison, Louise D. McCullough

**Affiliations:** Department of Neurology, University of Connecticut Health Center, 263 Farmington Avenue, Farmington, CT 06030 USA; Department of Immunology, University of Connecticut Health Center, Farmington, CT USA

**Keywords:** Microglia, Monocytes, Stroke, Inflammation, Phagocytosis

## Abstract

**Background:**

The brain’s initial innate response to stroke is primarily mediated by microglia, the resident macrophage of the CNS. However, as early as 4 h after stroke, the blood–brain barrier is compromised and monocyte infiltration occurs. The lack of discriminating markers between these two myeloid populations has led many studies to generate conclusions based on the grouping of these two populations. A growing body of evidence now supports the distinct roles played by microglia and monocytes in many disease models.

**Methods:**

Using a flow cytometry approach, combined with *ex-vivo* functional assays, we were able to distinguish microglia from monocytes using the relative expression of CD45 and assess the function of each cell type following stroke over the course of 7 days.

**Results:**

We found that at 72 h after a 90-min middle cerebral artery occlusion (MCAO), microglia populations decrease whereas monocytes significantly increase in the stroke brain compared to sham. After stroke, BRDU incorporation into monocytes in the bone marrow increased. After recruitment to the ischemic brain, these monocytes accounted for nearly all BRDU-positive macrophages. Inflammatory activity peaked at 72 h. Microglia produced relatively higher reactive oxygen species and TNF, whereas monocytes were the predominant IL-1β producer. Although microglia showed enhanced phagocytic activity after stroke, monocytes had significantly higher phagocytic capacity at 72 h. Interestingly, we found a positive correlation between TNF expression levels and phagocytic activity of microglia after stroke.

**Conclusions:**

In summary, the resident microglia population is vulnerable to the effects of severe ischemia, show compromised cell cycle progression, and adopt a largely pro-inflammatory phenotype after stroke. Infiltrating monocytes are primarily involved with early debris clearance of dying cells. These findings suggest that the early wave of infiltrating monocytes may be beneficial to stroke repair and future therapies aimed at mitigating microglia cell death may prove more effective than attempting to elicit targeted anti-inflammatory responses from damaged cells.

**Electronic supplementary material:**

The online version of this article (doi:10.1186/s12974-015-0329-1) contains supplementary material, which is available to authorized users.

## Introduction

Inflammation is a key component of stroke-induced injury and elevated levels of inflammatory markers are associated with poor outcome in stroke patients [1–4]. Inflammation in the brain is generally mediated by microglia, the resident macrophage of the CNS [5]. In the protected confines of the blood–brain barrier, microglia maintain healthy brain function by clearing debris, pruning synapses, and producing growth/repair factors [6]. These cells stand poised to respond to injuries in the CNS such as ischemic stroke. Numerous experimental studies have shown that microglia become activated following stroke, notably shifting their morphology from a thin, ramified state to a large, amoeboid structure [7]. This change is generally thought to be accompanied by an increase in proliferation and production of inflammatory mediators. Yet despite the evidence for widespread recruitment of bone marrow-derived monocytes (and their derivatives) to injured brain regions and a lack of discriminate cell markers, little is known regarding the functional differences between these two myeloid populations in ischemic stroke. Given the high degree of macrophage heterogeneity that comprises our innate immune system, the functional role of these populations is likely distinct and of translational importance [8–11]. CNS-resident microglia are the first responders to ischemia; these cells likely serve a unique role in injury repair relative to monocytes, which have a finite lifespan and are recruited in higher numbers during the post-reperfusion phase from the periphery. These differences have been made evident in recent studies that utilize transgenic bone marrow chimeras to distinguish between local and circulating myeloid populations [12]. These potentially disparate roles suggest that drugs designed to modulate microglia function may adversely impact that of the infiltrating monocyte population.

In this study, we investigated functional differences between brain-resident microglia and infiltrating monocytes in acute ischemic injury to better understand the contribution of each population to the recovery phase of stroke. Using flow cytometry to discriminate between these two populations, we applied *ex-vivo* functional assays to ascertain their functional roles during stroke and early recovery. By identifying the distinct function of microglia and monocytes early after ischemic stroke, the potential for targeting these specific cell populations will allow for the development of more effective therapeutic interventions.

## Materials and methods

### Mice/animals

Young adult C57BL/6 J male mice (10–12 weeks) of age were pair-housed on sawdust bedding in a pathogen free facility (light cycle 12/12 h light/dark). All animals had access to chow and water ad libitum. All procedures were performed in accordance with NIH guidelines for the care and use of laboratory animals and approved by the Institutional Animal Care and Use Committee of the University of Connecticut Health Center. All analyses were performed blinded to surgical conditions.

### Ischemic stroke model

Cerebral ischemia was induced by 90 min of reversible middle cerebral artery occlusion (MCAO, 20–25 gm mice) under isoflurane anesthesia as previously described [13]. Rectal temperatures were maintained at approximately 37 °C during surgery and ischemia with an automated temperature control feedback system. A midline ventral neck incision was made, and unilateral MCAO was performed by inserting a 6.0 Doccol monofilament (Doccol Corp, Redlands, CA, USA) into the right internal carotid artery 6 mm from the internal carotid/pterygopalatine artery bifurcation via an external carotid artery stump. Following reperfusion mice were sacrificed at 24 and 72 h and 7 days. Sham-operated animals underwent the same surgical procedure, but the suture was not advanced into the internal carotid artery.

### Tissue harvesting

Mice were euthanized, transcardially perfused with 60 mL cold, sterile PBS, and the brains were harvested. The brainstem, cerebellum, and olfactory bulbs were removed. The brain was then divided along the interhemispheric fissure into two hemispheres and subsequently rinsed with PBS to remove contaminant cells.

### Flow cytometry

Brains were placed in complete Roswell Park Memorial Institute (RPMI) 1640 (Lonza) medium and mechanically and enzymatically digested in collagenase/dispase (1 mg/mL) and DNAse (10 mg/mL; both Roche Diagnostics) for 1 h at 37 °C. The cell suspension was filtered through a 70 um filter. Leukocytes were harvested from the interphase of a 70 %/30 % Percoll gradient. Cells were washed and blocked with mouse Fc Block (eBioscience) prior to staining with primary antibody-conjugated flourophores: CD45-eF450, CD11b-APCeF780, Ly6C-PerCP-Cy5.5, Ly6G-PE, and SIRPα-APC. All antibodies were commercially purchased from eBioscience. For live/dead discrimination, a fixable viability dye, carboxylic acid succinimidyl ester (CASE-AF350, Invitrogen), was diluted at 1:300 from a working stock of 0.3 mg/mL. Cells were briefly fixed in 2 % paraformaldehyde (PFA). Data were acquired on a LSRII using FACSDiva 6.0 (BD Biosciences) and analyzed using FlowJo (Treestar Inc.). No less than 100,000 events were recorded for each sample. Resident microglia were identified as the CD45^int^ CD11b^+^Ly6C^−^ population, whereas bone marrow-derived leukocytes were identified as CD45^hi^CD11b^+^Ly6C^+^Ly6G^−^. Cell type-matched fluorescence minus one (FMO) controls were used to determine the positivity of each antibody. Prior to assessment on the cytometer, isolated cells were briefly probed to determine phagocytosis activity, oxidative stress level, cell proliferation status, and cytokine production as described below.

### Phagocytosis bead assay

To study the phagocytic activity of microglia, fluorescent latex beads (Fluoresbrite Yellow Green (YG) carboxylate microspheres; 1um diameter; Polysciences) were added to sorted microglia in a final dilution of 1:100 as described [14]. After 1-h incubation at 37 °C, the cells were washed three times with FACS buffer, re-suspended in FACS buffer, stained for surface markers, and fixed in PFA (*N* = 6/group). Mean fluorescence was determined from the YG bead + microglia population and used to measure the amount of beads per phagocytosing cell [15].

### Reactive oxygen species measurement

For detection of reactive oxygen species, microglial cells were incubated with redox-sensitive DHR (5uM; Ex/Em: 495/520) fluorogenic cell-permeant dye (Life Technologies, Invitrogen). Cells were incubated for 20 min at 37 °C, washed three times with FACS buffer (without NaAz), and then stained for surface markers including CASE (*N* = 5/group). After loading cells with DHR, each sample was separated into two tubes, one kept on ice and one at 37 °C.

### BRDU labeling and analysis

For cell proliferation studies, 50 mg/kg of BRDU (Sigma) was injected interperitoneally starting at 12 h after reperfusion and again at 24 and 48 h. BRDU staining was assessed using a BRDU Flow Kit (BD Biosciences). In brief, cells were permeabilized with detergent and treated with DNAse prior to the addition of both anti-BRDU-FITC antibody and Ki67-PE (eBioscience).

### Intracellular cytokine production

For intracellular cytokine staining, an in vivo brefeldin A (BFA) protocol was followed. Briefly, 10 mL/kg of BFA (Sigma, 0.5 mg/mL in DMSO) was injected via tail vein. Ten hours later, animals were sacrificed and tissue was harvested as noted above. Prior to staining, 1 ul of GolgiPlug containing brefeldin A (BD Biosciences) was added to 800 ul complete RPMI and cells were incubated for 2 h at 37 °C (5 % CO2). Afterward, cells were re-suspended in Fc Block, stained for surface antigens and washed in 100 ul of fixation/permeabilization solution (BD Biosciences) for 20 min. Microglia were then washed twice in 300 ul permeabilization/wash buffer (BD Biosciences), re-suspended in an intracellular antibody cocktail containing TNF-PE-Cy7 (eBioscience) and IL-1β-FITC (eBioscience) and subsequently fixed (*N* = 5-7/group).

### Bone marrow chimera generation

Wildtype C57BL/6 J mice (8 weeks old) were lethally irradiated (two doses of 5–6 Gy) in a Gammacell 40 research irradiator, and 5 × 10^5^ nucleated GFP-expressing donor bone marrow cells were injected retro-orbitally [16]. Chimeras were maintained on sulfamethoxazole/trimethoprim antibiotics in their drinking water 1 day prior and 2 weeks following irradiation. Chimeras were used for experiments 10 weeks following transplantation.

### Statistical analyses

Data from individual experiments are presented as mean ± SEM and assessed by Student’s *t* test or one-way ANOVA with Tukey post-hoc test for multiple comparisons (GraphPad Prism Software Inc, San Diego, CA, USA). For two-way ANOVA, significant differences between paired comparisons were conducted with the Holm-Sidak test. The Spearman’s rank correlation test was used to ascertain the correlation between cytokine production and phagocytic activity. Significance was set at *p* < 0.05.

## Results

### Ischemic stroke induces microglial death, bone marrow production of monocytes, and recruitment of monocytes to the injured brain

**Fig. 1:**
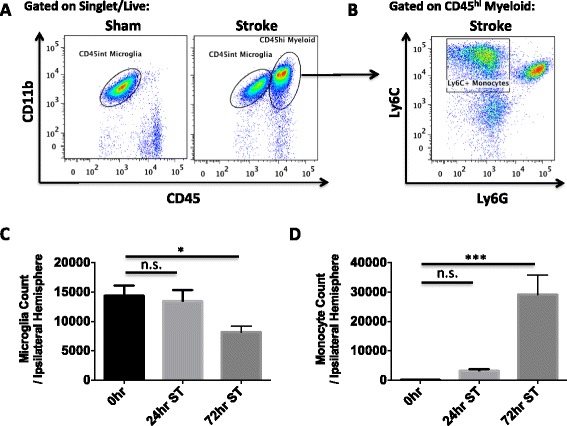
Number of microglia and monocytes in the ischemic hemisphere in the early period after stroke. A *representative dot plot* depicts the gating strategy used to identify both brain-resident microglia and Ly6C^+^ monocytes at 72 h after 90-min MCAO (**a**, **b**). Absolute cell counts of microglia (**c**) and monocytes (**d**) were quantified at 0, 24, and 72 h after stroke. For all experiments, *N* = 9/group. *Error bars* show mean SEM. Abbreviation: *SEM* standard error of the mean. *p < 0.05; ***p < 0.001

**Fig. 2:**
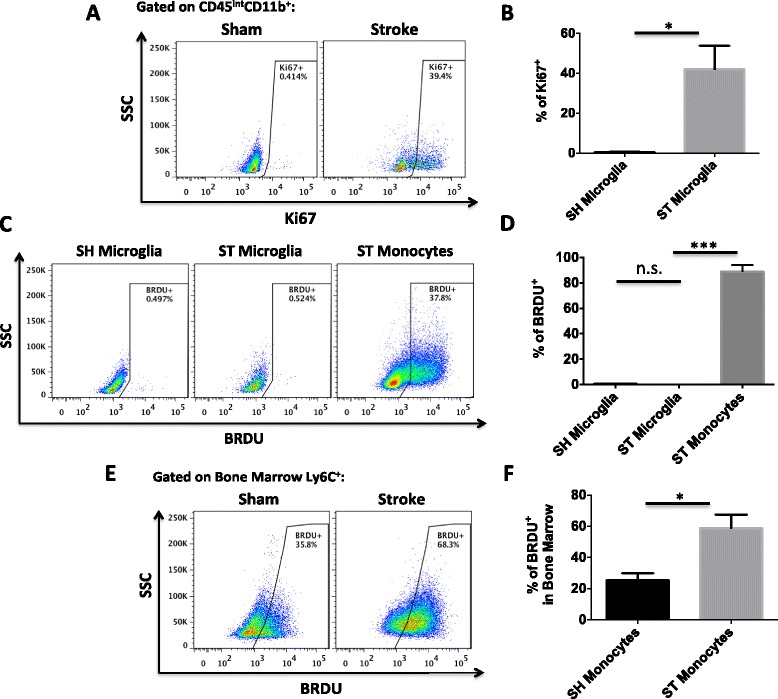
Assessment of microglia proliferation and monocyte production by 72 h after stroke. A *representative dot plot* shows Ki67 expression by microglia at 72 h after stroke (**a**). The percentages of Ki67^+^ microglia were quantified (**b**). BRDU incorporation of myeloid cells was assessed in the brain using flow cytometry. A *representative dot plot* illustrates that resident microglia had not undergone DNA synthesis by 72 h in the ischemic brain (**c**). Quantification of BRDU^+^ cells revealed that nearly all monocytes had entered S phase of proliferation (**d**). Analysis of bone marrow revealed that stroke-induced production of monocytes likely account for BRDU^+^ monocytes in the brain (**e**, **f**). For all experiments, *N* = 5/group. Cell-specific FMO controls were used to determine positive gating. *Error bars* show mean SEM. Abbreviation: *SEM* standard error of the mean, *SH* sham, *ST* stroke. *p < 0.05; ***p < 0.001

We confirmed the ability to reliably distinguish CD45^int^ microglia from CD45^hi^ monocyte populations in the ischemic brain by generating GFP bone marrow chimeras, in which all bone marrow-derived cells were GFP-positive (Additional file 1: Figure S1A). We demonstrated that the two populations did not significantly overlap after stroke, validating this approach. Absolute leukocyte counts were obtained by flow cytometry at 24 and 72 h after MCAO in non-irradiated, intact wild type mice using the gating strategy shown in Fig. 1a, b. A significant reduction in the number of microglia (CD45^int^CD11b^+^Ly6C^−^) after stroke was found after 24 h (*p* < 0.05; Fig. 1c). Conversely, we found a dramatic increase in monocyte (CD45^hi^CD11b^+^Ly6C^+^Ly6G^−^) counts in the stroke hemisphere compared to sham brain (Fig. 1d). At 72 h, microglia expressed increased levels of Ki67, a marker of actively cycling cells (*p* = 0.017; Fig. 2a, b). DNA synthesis, an indicator of cell proliferation, was then measured by BRDU incorporation. Following repeated BRDU injections starting at 12 h, microglia showed little BRDU incorporation by 72 h, whereas ~90 % of monocytes in the ischemic brain were BRDU-positive (Fig. 2c, d). Stroke is known to stimulate bone marrow production of myeloid cells that are subsequently recruited to the brain [17]. We found a significant increase in the percentage of BRDU^+^ monocytes in the bone marrow following stroke (*p* = 0.016; Fig. 2e, f). These data suggest that after 90-min tMCAO, there is a significant loss of resident microglia, impaired cell cycle progression, and an increased number of newly produced, bone marrow-derived monocytes.

### Differential oxidative stress and cytokine production by microglia and monocytes after stroke

**Fig. 3:**
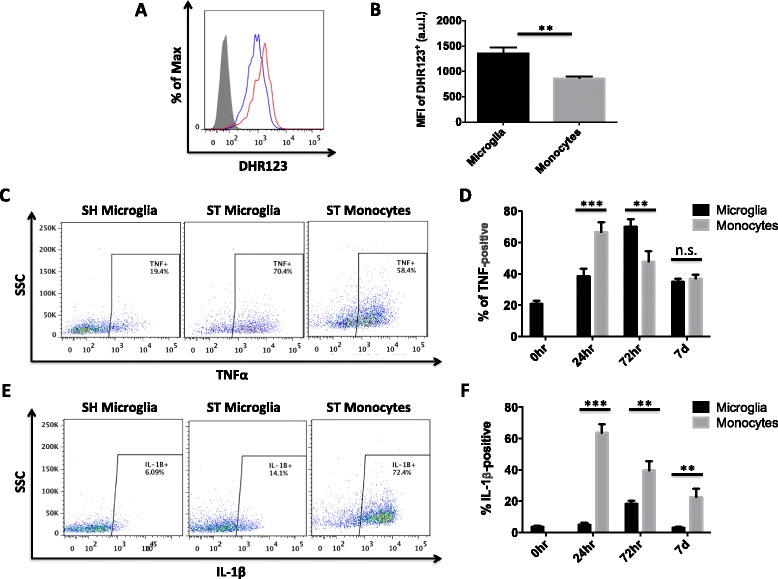
Measurement of cytokine production by microglia and monocytes at 24 and 72 h and 7 days following stroke. A representative histogram showing microglia (*red*) have relatively higher reactive oxygen species levels than monocytes (*blue*) at 72 h after stroke as measured with dihydrorhodamine (DHR) 123 (**a**). The mean fluorescence intensity of DHR123 was quantified (**b**). *Representative dot plots* showing microglia and monocyte production of TNF (**c**) and IL-1β (**e**) at 72 h reveals differential expression patterns after stroke. The respective percentages of cells positive for these cytokines are quantified at 0, 24, and 72 hrs and 7 days (**d**, **f**). For all experiments, *N* = 5–7/group. Cell-specific FMO controls were used to determine positive gating. *Error bars* show mean SEM. Abbreviation: *SEM* standard error of the mean, *SH* sham, *ST* stroke, *MFI* mean fluorescence intensity. **p < 0.01; ***p < 0.001

Reactive oxygen species production drives the oxidative stress response to stroke and is critical to injury progression [18]. We examined free radical formation in microglia and monocytes after stroke. Resident microglia expressed significantly more ROS relative to the infiltrating monocytes (*p* < 0.01), as evidenced by increased DHR123 staining intensity (Fig. 3a, b). Because oxidative stress precipitates cytokine production, we next evaluated pro-inflammatory cytokine levels in these cells. Newly recruited monocytes expressed higher levels of TNF at 24 h; however, microglia expressed significantly greater levels by 72 h (Fig. 3c, d). Infiltrating monocytes expressed relatively higher levels of IL-1β at all time points (*p* < 0.001; Fig. 3e, f). The number of microglia expressing TNF remained elevated above sham levels at 7 days, whereas those expressing IL-1β did not differ. Neither microglia nor brain-recruited monocytes expressed detectable levels of the anti-inflammatory cytokines IL-4 and IL-10 after stroke (data not shown). These results suggest that microglia play a critical role in oxidative injury and that these two cell types may mediate cell death via distinct signaling pathways.

### Monocytes are the predominant phagocytes in the brain at 72 h after stroke

**Fig. 4:**
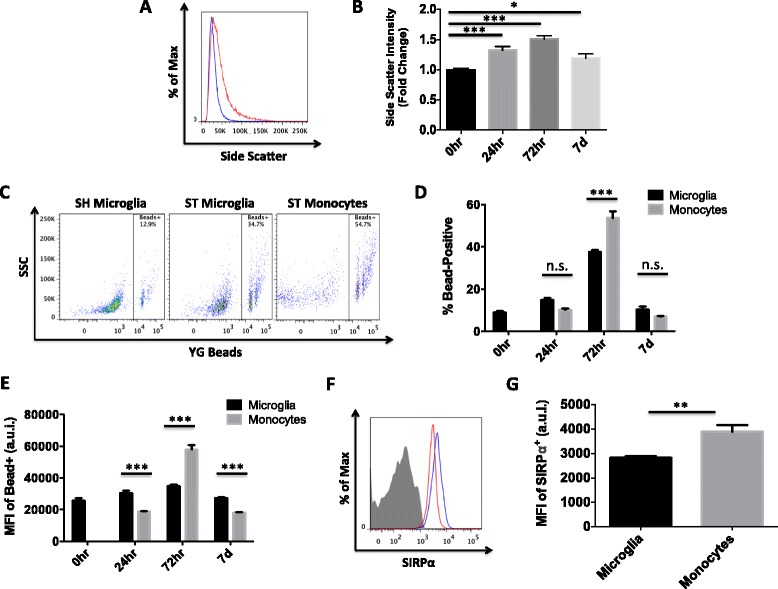
Phagocytic activity of microglia and monocytes at 24 and 72 h and 7 days following stroke. Representative histogram showing a relative increase in side scatter (granularity) properties of microglia at 72 h after stroke (*red*) compared to sham (*blue*; **a**). Mean side scatter intensity of microglia was quantified at different time points after MCAO (*N* = 5/group; **b**). Phagocytic activity after stroke was measured by bead assay using flow cytometry (**c**). The percentages and mean fluorescence intensity (MFI) of bead^+^ cells were quantified at 0, 24, and 72 h and 7 days (*N* = 6/group; **d**, **e**). A representative histogram shows higher expression of the phagocytic marker SIRPα on monocytes (*blue*) compared to microglia (*red*) at 72 h in the ischemic hemisphere (**f**). The MFI of SIRPα^+^ cells are quantified (*N* = 5/group; **g**). Cell-specific FMO controls were used to determine positive gating. Error bars show mean SEM. Abbreviation: *SEM* standard error of the mean, *SH* sham, *ST* stroke, *SSC* side scatter, *YG* yellow green, *a.u.i.* arbitrary units of intensity

**Fig. 5:**
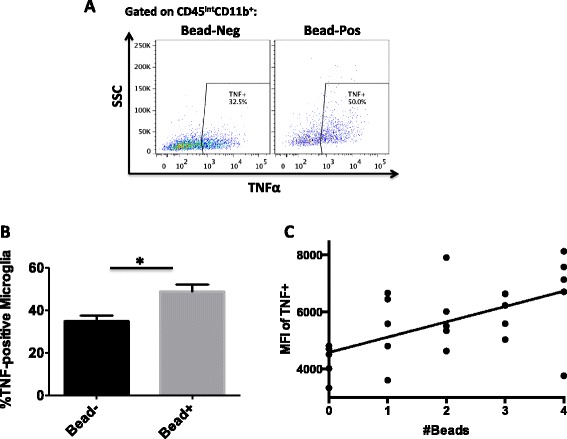
Relationship between TNF production and phagocytic activity of microglia after stroke. A *representative dot plot* shows the relationship between microglial TNF production and phagocytic activity at 24 h (**a**). The percentage of TNF^+^ microglia is significantly higher in the phagocytic subset of microglia compared to the non-phagocytic subset (*N* = 5/group; **b**). A positive correlation was found between the MFI of TNF expression in microglia and number of beads they phagocytosed after stroke (**c**). Cell-specific FMO controls were used to determine positive gating. *Error bars* show mean SEM. Abbreviation: *SEM* standard error of the mean, *SSC* side scatter, *MFI* mean fluorescence intensity, *a.u.i.* arbitrary units of intensity. *p < 0.05

Microglia in the ischemic hemisphere were significantly more activated based on side scatter (granularity) properties compared to sham, suggesting enhanced uptake of dying cells and debris (Fig. 4a, b). Phagocytosis is important in debris clearance and injury repair. Using a bead assay, we measured phagocytic activity of microglia and monocytes (Fig. 4c). After stroke, the percentage of microglia that phagocytosed beads significantly increased at 24 h (*p* < 0.001) and peaked at a near four-fold increase by 72 h (*p* < 0.01; Fig. 4d). The phagocytic activity of microglia is restored back to baseline levels by 7 days. Compared to microglia, however, infiltrating monocytes exhibited far greater capacity for phagocytosis. Significantly more monocytes were bead-positive as were the number of beads phagocytosed per cell as evidenced by MFI (*p* < 0.001; Fig. 4e). Monocytes at 72 h also expressed significantly higher levels of the phagocytic marker SIRPα than did microglia (*p* = 0.005; Fig. 4f, g). Interestingly, phagocytic microglia were more likely to express TNF and at higher levels than non-phagocytic microglia, indicating that pro-inflammatory (M1) markers may overlap with anti-inflammatory (M2) function (Fig. 5a, b). Moreover, a positive correlation was found between the level of TNF production and the number of beads phagocytosed by microglia after stroke (*p* = 0.0167; Fig. 5c). Taken together, these data imply that microglia do increase phagocytic activity following stroke stimulus, albeit at significantly lower levels than recruited monocytes.

## Discussion

We demonstrate for the first time that the increase in macrophage proliferation in the brain following severe tMCAO is a result of stimulus-driven bone marrow production of migrant monocytes rather than proliferation of microglia. Resident microglia, as bystanders to ischemia, are similarly vulnerable to its detrimental effects. We found that after a 90-min MCA occlusion, the number of microglia is significantly less than that seen in sham brain. Traditionally, the use of standard immunohistochemistry could not make this distinction; possibly overestimating the microglial response leads to the concept that microglia are highly proliferative, even after severe ischemic injuries. Indeed, the microglia population in general appears to be severely compromised after stroke. It is not known whether the microglial population replenishes itself during recovery or if invading monocytes establish permanent residence and take over the microglial niche [19, 20].

Although microglia and monocytes exhibit several features of pro-inflammatory activation after stroke, several studies suggest a supportive and even beneficial role for each in stroke outcome [21–27]. These outcomes are likely influenced by the degree of ischemic severity and the ischemic setting/conditions. Distinguishing brain-resident microglia from monocyte populations in ischemic brains that have high numbers of infiltrating leukocytes can be challenging. Due to the lack of available microglia-specific markers, the absolute identification of microglia can be hampered by up-regulation of CD45 expression on activated microglia after injury, which can cause overlap with bone marrow-derived macrophage populations [28–30]. Bone marrow chimeras allowed us to differentiate between all fluorescently tagged bone marrow-derived leukocytes from resident microglia, definitively confirming that microglial expression of CD45 is relatively stable after stroke. However, to further ensure the purity of our microglial gating strategy, we selected out any contaminating monocytes using Ly6C antibody, which is not expressed on adult microglia. These findings enabled us to provide a reliable functional assessment of resident microglia and recruited monocyte populations by *ex-vivo* flow cytometry.

Recent work from the Merad laboratory has elegantly demonstrated that there is little bone marrow or systemic contribution to the resident microglia pool under normal conditions [31, 32]. These observations were made using donor-tagged bone marrow chimeras and parabiotic mice. Consistent with these findings, our group has also found that as far out as 1 year following radiation, uninjured chimeras had less than 5 % “microglia-like” monocytes as evidenced by their CD45^int^CD11b^+^GFP^+^Ly6C^+^ phenotype, reminiscent of the resident CD45^int^CD11b^+^GFP^−^ microglia population (data not shown). Although these findings imply there is a low frequency of monocyte mixing in the resident CD45^int^ microglia population of irradiated chimeras, these bone marrow-derived GFP^+^ cells can still be distinguished by the expression of Ly6C. Although useful, chimera development and parabiotic manipulation have limitations, including the potential for radiation injury and immune activation from the invasive surgical stress, respectively. Importantly, other studies have found that following an injury to the CNS, recruited monocytes can down-regulate Ly6C expression and establish permanent residence, thereby replenishing the attenuated microglia pool [19, 33]. The acute time points described in our study are unlikely to be affected by the down-regulation of Ly6C on monocytes as earlier studies have shown that this does not occur until after 7 days post-injury. While the genotoxic elimination of the microglia in mice appears to be non-deleterious in the short-term, the long-term consequences of microglia removal are likely detrimental to CNS homeostasis as evidenced in several neurodegenerative models [34–37]. The long-term consequences of microglia elimination in necrotic regions following ischemic stroke warrants further investigation.

We show that microglia numbers are decreased at 72 h after a 90-min tMCAO and noted an inverse correlation between microglia and infiltrating monocyte counts. This is in contrast to several reports that have shown significant increases in microglia proliferation [38–40]. However, it is likely that the severity of the ischemic injury plays a major role in the microglial response as other work has shown reduced microglial populations with more severe injury (e.g., longer occlusion times) [41]. Previous work has shown that the morphological changes that accompany glial cell activation after stroke are not dependent on or coincident with neuronal death but rather the duration of ischemia [42]. Microglia are more ischemia-resistant than neurons and oligodendrocytes, but clearly, they are also vulnerable to severe ischemia [43–45]. The inability to distinguish microglia from infiltrating monocytes, which increase in number with injury severity using histological methods, may further obfuscate this issue. Flow cytometry may provide a more sensitive means of identification of cell type based on the well-accepted relative surface expression of CD45. Depending on the compensation setup, overlapping of these cell populations may also occur with flow cytometry. However, we have shown that it is extremely useful to use Ly6C as a monocyte marker to achieve maximal separation of these two populations, as infiltrating monocytes may down-regulate CD45 and MHCII levels over time, adopting a microglia-like phenotype (CD45^int^CD11b^+^) [33].

Increased production of bone marrow-derived myeloid cells after stroke has been described [17]. We found that BRDU is incorporated into many newly produced monocytes in the bone marrow, which are subsequently recruited into the brain. This is consistent with recent work showing ischemic stroke activates hematopoietic stem cells in the bone marrow, resulting in a myeloid bias and greater output of monocytes [46]. The number of BRDU-positive myeloid cells in the brain reaches its peak by 72 h [38, 41]. This parallels the time course of leukocyte infiltration into the brain, which also peaks at 72 h. Although microglia did not enter S phase in our study, many were Ki67^+^, indicating that they were actively cycling at 72 h. Ki67 expression in activated microglia after stroke has also been documented [47]. The failure of most cells to progress through later stages of the cell cycle may also be due to altered length of G1 and/or cellular stress [48]. Based on this earlier work and given the differential effects of ischemia on microglia populations located throughout the hemisphere, we hypothesized that some of these cells would enter the S phase of cell cycle to begin dividing prior to 72 h. Surprisingly, we did not observe this trend. An earlier study using GFP bone marrow chimeras revealed that the vast majority of macrophages in the infarct area after a 30-min tMCAO were derived from local microglia [49]. Despite the smaller injuries and altered leukocyte kinetics that result from shorter occlusion times, the ability to assess microglia numbers after stroke in an unbiased fashion using immunohistochemistry can be challenging due to the subtle difficulty of identifying microglia in their ramified state relative to those with amoeboid morphology and given the rapid migration of activated microglia into the penumbral region often present in the field of view. As such, shifts in population densities due to migration towards injury sites could be misinterpreted as local microglia proliferation. It should be noted, however, that our data do not exclude the possibility that microglia proliferation occurs beyond 72 h in the 90-min tMCAO model.

A recent study of microglia proliferation after stroke was elegantly approached by implementing the parabiosis model using wild type and CX3CR1^GFP/−^ mice [50]. The authors concluded that microglia proliferation accounted for the majority of microgliosis observed after stroke, rather than monocyte infiltration. However, there are several possible explanations for the disparate results. The CX3CR1-GFP signal in these reporter mice is not specific only to microglia, especially following an injury stimulus, as it has been shown that Ly6C^lo^ monocytes also express GFP [30, 51, 52]. As histological analyses would not be able to distinguish GFP^+^ microglia from GFP^+^ monocytes (or vice versa in non-GFP WT mice), these cells largely go undetected as they typically begin migrating to the brain within 12–24 h of injury. In addition, this study used a smaller, more localized injury induced photothrombosis, resulting in a milder inflammatory response. The stimulus-driven production of monocytes in bone marrow in this milder injury would likely be less and further diluted following egress into the blood due to the ~50 % equilibrium of circulating cells in parabiotic (GFP-WT) mice. These studies provide valuable information in the context of both the specific model of stroke being employed, the severity of ischemia, and the time points being investigated. Taken together, these authors nicely demonstrate that microglia proliferation occurs early after stroke under mild or modest ischemic conditions, whereas our data implies this function is likely compromised after severe ischemic injury.

Oxidative stress and cytokine-induced cell death are key components of ischemic injury. Reperfusion after ischemia produces a burst in ROS formation [53]. Oxidative stress increases within the first hour after reperfusion and delivers signals that promote necrosis and apoptosis. The generation of ROS burst activity results from impaired mitochondrial respiratory chain function and activation of cytoplasmic oxidases. As a result, oxidative stress contributes directly to necrosis and apoptosis through a number of pathways in ischemic tissue. Although neurons and endothelial cells are important sources of ROS, all cells subject to ischemic injury are vulnerable to ROS-mediated cell death signaling [54]. The massive influx of ROS producing infiltrating leukocytes adds considerably to the overall ROS levels in the brain after stroke. Using bone marrow chimeras, a recent study demonstrated that NADPH oxidase (NOX2)-mediated ROS production in circulating leukocytes contributed to exacerbated infarct volumes compared to that produced by resident microglia after stroke [55]. Although we found that ROS levels were higher in microglia after stroke, we did not evaluate the other subsets of infiltrating myeloid cells, namely neutrophils. Instead, we show that on cell-to-cell basis, monocytes produce significantly less ROS after stroke than resident microglia. This may reflect the severely compromised state of microglia following ischemic reperfusion.

Injury-driven production of ROS can further amplify the inflammatory response by driving cytokine production. TNF and IL-1β are produced by both microglia and monocytes after stroke. Numerous studies using transgenic mice and pharmacological agents have demonstrated a neurotoxic role for each of these pro-inflammatory cytokines in ischemic injury [56, 57]. Our finding that microglia produced higher levels of ROS and TNF after stroke than monocytes suggest that the resident cells may be the more detrimental macrophage early after injury. Excessive insult to resident microglia may impair their primary function to maintain brain homeostasis during recovery. These findings build on previous work which described the expression of these cytokines in different subsets of microglia and macrophages 24 h after permanent MCAO using a bone marrow chimera approach combined with immunohistochemistry and flow cytometry [58]. The authors reported that a greater number of CD11b^+^ CD45^hi^ macrophages/granulocytes expressed TNF and IL-1β at 24 h compared to CD11b^+^ CD45^dim^ microglia. While innovative in its design, the use of GR-1 to distinguish between and identify monocytes/macrophages and neutrophils is not as accurate as Ly6C/Ly6G markers [51]. Despite this discrepancy, we found similar expression profiles in these two populations at 24 h, whereas microglia became the predominant TNF producers by peak leukocyte infiltration at 72 h. The importance of TNF signaling to ischemic injury was recently evaluated [59]. Using bone marrow chimeras, it was found that TLR2- and TLR4-mediated injury is primarily driven by infiltrating leukocytes rather than resident microglia. However, TNF is also produced by microglia early after ischemia prior to monocyte infiltration, and TNF-directed therapies at these early time points are neuroprotective [60, 61]. However, it must also be noted that infiltrating monocytes expressed higher levels of IL-1β, a cytokine associated with inflammasome signaling [62, 63]. This implies that inflammatory signaling in macrophage populations is incredibly robust and complex. Further understanding of these differences between these two cell subtypes and their inflammatory function after stroke may provide better insight on the targeted effects of known drug treatments, allowing for the development of more effective, cell-specific drugs.

Following ischemia, injured areas of the brain undergo apoptosis. Dead and dying cells generate a vast amount of debris that requires clearance for regenerative processes to ensue. As the ischemic/reperfusion injury core evolves after 24 h, amoeboid microglia migrate from the core to the transition zone of the penumbral area following reperfusion and adopt an M1 phenotype [64, 65]. The importance of debris clearance by phagocytes is evident in rodents and humans lacking the scavenger receptor gene CD36, resulting in exacerbated injury and hematoma absorption in models of ICH [66, 67]. MRI imaging has demonstrated that USPIO particles can be tracked and localized to ED1+ microglia/macrophages with the highest signal intensities found in striatal and cortical penumbral regions at day 2 after 30 min tMCAO in rats [68]. Using GFP bone marrow chimeras, an earlier study had shown that activated microglia phagocytose neuronal material as early as 24 h and are the predominant phagocyte by day 7 [69]. While an early preponderance of phagocytic microglia would be expected given the delayed recruitment of monocytes, their use of a less severe, 30-min tMCA occlusion resulted in attenuated numbers of infiltrating monocytes. In another study, rats that had undergone a 3-hr tMCAO exhibited poor microglial phagocytosis of caspase-3-expressing neurons at 24 h [24]. Moreover, the number of apoptotic neurons was not increased following liposome-mediated ablation of microglia, indicating a limited contribution to debris clearance in environments that are significantly impacted by ischemia. Injury-related defects in migration and increased cytokine repulsion signals may halt microglia phagocytic activity under increasing ischemic stress.

We provide the first functional evidence that microglia increase capacity for phagocytosis in acute ischemic stroke. The number of phagocytic microglia initially increased at 24 h, peaked at 72 h, and returned to baseline by 7 days. These data paralleled the increase in microglia side scatter properties (granularity) in separate experiments, further indication of phagocytic uptake. Interestingly, however, infiltrating monocytes were significantly more efficient at phagocytosing material during the peak response at 72 h. Following tissue entry, monocytes may further mature into macrophage or dendritic cell subsets with tissue-specialized functions. Monocyte-derivatives retain the expression of lineage markers and have enhanced phagocytic ability [70]. Our findings indicate that while these tissue phagocytes exhibit many classical pro-inflammatory features deemed destructive, they appear to play a critical role in debris clearance and post-stroke recovery. It may be that in addition to being injured, many resident microglia had already exhausted their capacity to phagocytize surplus debris prior to *ex-vivo* phagocytosis assessment. However, by 7 days after stroke, when monocytes diminish in the ischemic brain, resident microglia again become the predominant phagocyte, highlighting the importance of microglia in the long-term recovery and repair process. Unpublished results from our laboratory suggest that circulating phagocyte populations are generally more efficient than microglia at phagocytizing material under non-stimulated conditions. Recent work demonstrating the importance of monocytes to hemorrhage clearance in a model of intracerebral hemorrhage highlights their ability to significantly improve long-term outcomes through phagocytic function early after stroke [71]. This may relate in part to the degree of adaptive functionality associated with their different life-spans, as monocyte function may be more robust given their “boom and bust” half-life, whereas the phagocytic activity of microglia likely endures at a steady rate throughout life. It is important to note that monocyte-targeted therapies aimed at inhibiting the function of these cells may inadvertently affect their phagocytic activity. Moreover, strategies to exploit this capacity should be considered, especially during the early stages of injury when the blood–brain barrier is susceptible to monocyte entry.

Finally, we showed a positive correlation between TNF expression levels and the level of phagocytic activity of microglia after injury. ROS and pro-inflammatory cytokine signaling is thought to hamper phagocytic activity and debris clearance [72, 73]. Our finding suggests that dichotomizing function into M1/M2 polarization, in which cells are polarized to become either pro-inflammatory/detrimental (M1) or anti-inflammatory/injury resolving (M2) may be too simplistic [74]. This concept more aptly applies to microglia in vitro and its polarization may be better appreciated as a continuum of activation in vivo due to the combination of factors and diverse cell types present in intact tissue. Indeed, the polarization dynamics of microglia and monocytes during stroke appear to be mixed and complex. As mentioned, anti-inflammatory markers such as IL-4 and IL-10 were not expressed by either myeloid cell at any time point in this study. However, we acknowledge that transitioning between M1 and M2 phases may occur weeks after injury as has been reported by others [5, 65]. Whether there is a requirement for pro-inflammatory signaling to induce or enhance phagocytic activity and debris clearance is currently not known. These findings point to a possible side effect of microglia/macrophage inhibition that could hamper the removal of dead cells and slow recovery.

In conclusion, we have identified distinct functional roles for brain-resident and infiltrating macrophages after stroke. To better both the interpretation of failed clinical trials and the development of more efficacious drugs to treat sterile inflammation in the CNS requires a better understanding of potential cellular targets. We believe that functional assessment of immune cells after stroke *ex-vivo* could be a powerful tool in screening and validating the targeted cellular effects of drugs and various therapeutic interventions.

## Additional file

Additional file 1: Figure S1. Relative stability of CD45 expression by microglia at 72 hrs after stroke. Bone marrow chimeras were generated using GFP^+^ donors and wildtype recipient mice. Microglial CD45 expression level was evaluated to determine if their CD45-intermediate expression level is increased after stroke, potentially overlapping with the CD45-high expressing bone marrow-derived myeloid population. A representative dot plot of the ischemic brain at 72 hrs illustrates that GFP^−^ (wildtype) microglia do not substantially increase CD45 expression to CD45-high levels associated with GFP^+^ bone marrow-derived populations (98 % GFP reconstitution in this wildtype host; S1A). Cell-specific FMO controls were used to determine positive gating.

## Abbreviations

BRDU: bromodeoxyuridine
CNS: central nervous system
DHR123: dihydrorhodamine 123
FMO: fluorescence minus one
GFP: green fluorescent protein
IL-1β: interleukin-1 beta
MFI: mean fluorescence intensity
ROS: reactive oxygen species
SH: sham surgery
SIRPα: signal-regulatory protein alpha
SSC: side scatter
ST: stroke surgery
TNF: tumor necrosis factor
tMCAO: transient middle cerebral artery occlusion
