# Supplementary figures and images for: Functional differences between microglia and monocytes after ischemic stroke
Source: J Neuroinflammation. 2015 May 29;12:106. doi: 10.1186/s12974-015-0329-1 (PMC4465481; doi:10.1186/s12974-015-0329-1)

**A**

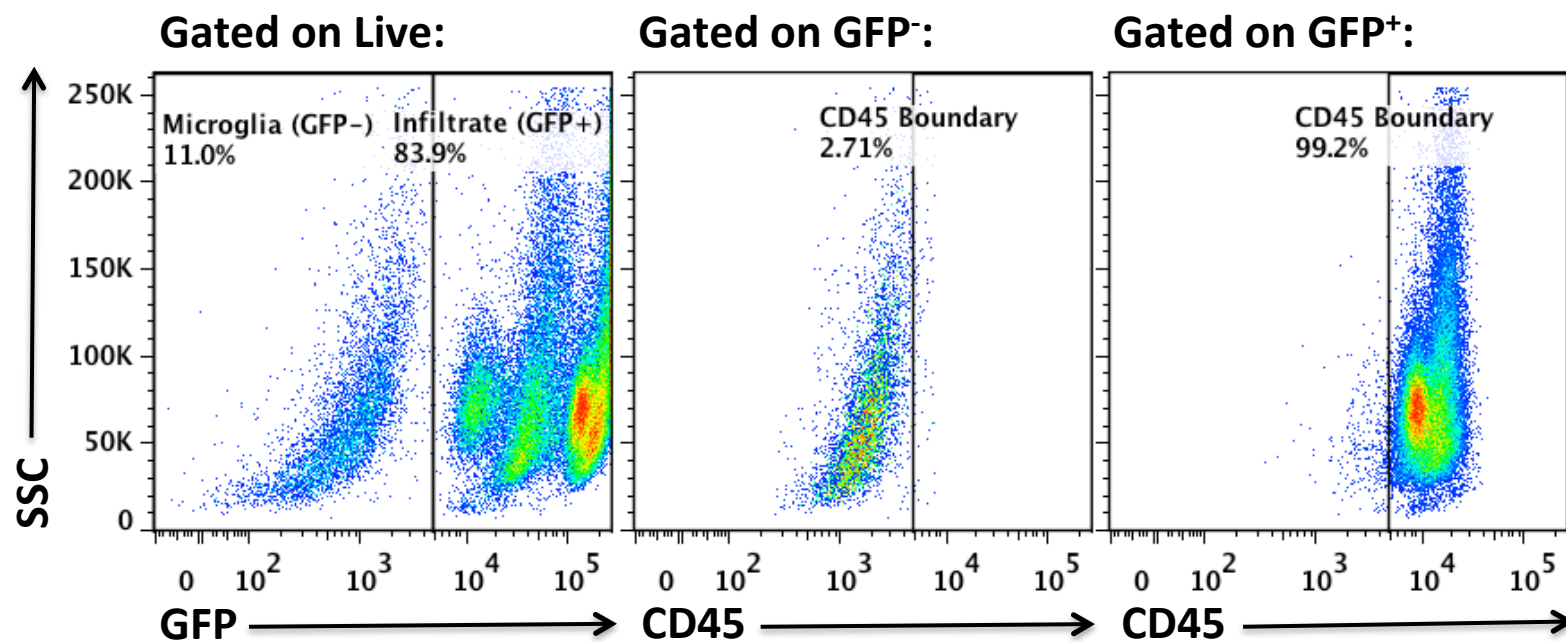

Supplement: Additional file 1: Figure S1. — Relative stability of CD45 expression by microglia at 72 hrs after stroke. Bone marrow chimeras were generated using GFP+ donors and wildtype recipient mice. Microglial CD45 expression level was evaluated to determine if their CD45-intermediate expression level is increased after stroke, potentially overlapping with the CD45-high expressing bone marrow-derived myeloid population. A representative dot plot of the ischemic brain at 72 hrs illustrates that GFP− (wildtype) microglia do not substantially increase CD45 expression to CD45-high levels associated with GFP+ bone marrow-derived populations (98 % GFP reconstitution in this wildtype host; S1A). Cell-specific FMO controls were used to determine positive gating. [file 12974_2015_329_MOESM1_ESM.pdf]
